# Ultrasound reliability for biliary atresia diagnoses in children: a single-center study

**DOI:** 10.5339/qmj.2025.68

**Published:** 2025-08-17

**Authors:** Ben Youssef Sabrine, Amani N. Al-Ansari, Maatouk Mezri, Ben Abdallah Ahmed, Mani Salma, Ben Fredj Myriam, Ben Salah Radhouane, Abdelaaly Mabrouk, Ksia Amine, Zrig Ahmed, Sahnoun Lassad

**Affiliations:** 1 Pediatric Surgery Department, Fattouma Bourguiba University Hospital—LR12SP13—University of Monastir, Monastir, Tunisia; 2 Department of Pediatric Surgery, Hamad Medical Corporation, Doha, Qatar; 3 Radiology Department, Fattouma Bourguiba University Hospital—LR18SP08—University of Monastir, Monastir, Tunisia *Email: AAlansari9@hamad.qa

**Keywords:** Biliary atresia, infant cholestasis, neonatal jaundice, postnatal cholestasis, reliability, ultrasound

## Abstract

**Background:**

Biliary atresia (BA) is a lfe-threatening neonatal liver disease characterized by inflammation and obstruction of bile ducts. Identifying the most reliable and valid ultraaound (US) parameter or combination of parameters can enhance noninvasive diagnosis accuracy, potentially reducing unnecessary surgeries and treatment delays. We aimed to detect the mosi reliable and valid US parameter or combination of parameters to diagnose BA, thereby minimizing unnecessary surgical exploration or treatment delays.

**Methods:**

We conducted a prospective cohort study on cholestatic pediatric patients. Data were nollected between January 2013 and July 2019 from the Fattouma Bourguiba University Hospital Monastir, Tun isia, in callaboration with the pediatric surgical and radiological departments. After full clinical and laboratory evaluation, abdominal ultrasonography was performed to assess the biliary structure, with three possible outcomes: BA ruled out, confirmed, or inconclusive. Accordingly, the patients were divided into group 1 (diagnosed with BA) and group 2 (diagnosed with other biliary conditions).

**Results:**

The participants, 61 cholestatic neonates and infants,were aged 6 to 160 postnatal days (mean, 58.28 ± 34.24 days); 60.7% were male (*n* = 37). The gallbladder (GB) was not detected usig, US in 20 patients. BA diagnosis correlated with cord sign [X^2^ (1, *n* = 61) = 53.52], GB evacuation alteration [X^2^ (1, *n* = 61) = 18.41%], and hepatic artery/portal vein ratio [X^2^ (1, *n* = 61) = 30.25; normal value <0.49]. A positive cord sign or the presence of alteration of evacuation indicates the possibility of BA with a 100% sensitivity and 86.2% specificity. Similarly, with the presence of evacuation alteration or porta hepatic thickness (≥2.4), the sensitivity reached 100%; however, a specificity of 86.2% was observed.

**Conclusions:**

US parameters can detect patients with BA with approximately 100% sensitivity. However, additional research is needed to confirm negative cases. Multicenter studies are needed to verify our findings.

## INTRODUCTION

Biliary atresia (BA) is a rare congenital disease that typically appears at or shortly after birth. Its pathological aspects are characterized by obliterative cholangiopathy due to progressive fibrosis, which impacts both intrahepatic and extrahepatic bile ducts.^1,2^ The natural course of the disease progression involves hepatic biliary cirrhosis and end-stage liver failure,^3^ with an increased risk of death by the age of 3 years.^4^ Therefore, early and prompt diagnosis is mandatory for proper intervention to reduce complication risks.^5^ However, distinguishing the identification of BA from other less serious conditions presents a clinical dilemma and requires recourse to clinical, laboratory, and imaging techniques.^6^

Abdominal ultrasound (AUS) is a fundamental tool for distinguishing BA cases^7^ from other cases of nonsurgical conjugated jaundice. It ensures timely intervention, usually using the Kasai procedure.^8^ Postnatal imaging features needed for BA diagnosis are considered more challenging. However, despite the high sensitivity and specificity of a triangular cord thickness (TCT) sign in BA diagnosis, the procedural accuracy is highly variable (29%–100%) due to operator dependency.^9^ Therefore, an experienced US operator is crucial.^10,11^ However, gall bladder (GB) features appear earlier than TCT. A review reported that micro- and macro-cysts or polysplenia syndrome are highly specific to BA. Consequently, a patient with pale or acholic stools with signs of dilated liver bile ducts is unlikely to have BA.^12^ However, a normal US result does not rule out BA, which is characterized by the invisibility of the GB lumen, small size, irregular form, and no GB emptying after meals.^11,12^ Despite the relevance of abdominal US in the early and prompt diagnosis of BA, its sensitivity and specificity parameters remain unclear. We aimed to evaluate US performance in BA diagnosis and investigate the primary biliary and vascular abnormalities that are discovered during US examination.

This study aimed to identify the most sensitive and specific US parameters for early BA diagnosis to minimize unnecessary surgeries and facilitate timely, appropriate treatment. Additionally, the cost-effectiveness of US parameters compared to conventional computed tomography (CT) makes it an ideal choice for low-income medical facilities.

## PATIENTS AND METHODS

### Study design and setting

This prospective cohort study, conducted from January 2013 to July 2019 at Fattouma Bourguiba University Hospital in Monastir, Tunisia, aimed to reduce unnecessary neonatal and infant surgeries by evaluating reliable US parameters for early BA diagnosis. As a tertiary care provider with a 52-bed Pediatric Surgical Department, the hospital supported this work, which serves as a foundation for developing a BA diagnostic score. Conducted in a low-income setting, our findings advocate for the cost-effective use of hepatobiliary US as a diagnostic tool for BA.

### Population

All children admitted for investigation of cholestasis at the pediatric surgical department were included in the study. Parental informed consent was obtained before the commencement of any intervention. A pediatric hepatobiliary radiologist with at least 10 years' experience performed US examinations on all patients.

The patients were clinically examined, and full laboratory investigations were conducted, with US being the main imaging study. Notably, surgical exploration was performed when a BA diagnosis was confirmed or the precise diagnosis was inconclusive.

### Data collection

The US examination was performed using LOGIQ E9 (GE Healthcare, Milwaukee, WI), with the ML-6-15 linear array probe after a fasting period of ≥10 hours and after a meal. The GB was visualized based on subcostal parasagittal sections. Upon visualization of the GB, its shape, size, and volume were used as measures. GB evaluation and its integrity were assessed and classified according to Ohi anatomical classification. The Ohi classification for BA categorizes the disease into three types based on the location of the bile duct obstruction. Type I involves atresia of the common bile duct, accounting for approximately 10% of cases. Type II is characterized by atresia of the common bile duct and hepatic ducts, which is less common, with an incidence of around 2%. The most prevalent type, Type III, involves atresia of the extrahepatic bile ducts, including the porta hepatis, and represents about 88% of cases. This classification aids in determining the extent of the disease and tailoring appropriate treatment strategies.^13^ Furthermore, GB volume was reassessed for all patients in whom it was visualized 30 minutes after feeding. The cord sign was sought by measuring the thickness of the hilar plate. This corresponds to a hyperechoic zone located anterior to the right portal branch, just below the bifurcation. Vascular modifications were assessed based on the hepatic artery and portal vein diameters at the hepatic hilum. Measurements were taken from the inner wall to the inner wall, excluding the vessel walls and perpendicular to the vessel wall, to calculate the hepatic artery-to-portal vein (HA/PV) ratio. The cutoff values for various parameters were as follows: portal vein <2.4 mm, HA/PV <0.49 mm, hepatic artery <2.15 mm, GB volume ≤0.23 mL, GB length ≥17.5 mm, and GB width ≤4.5 mm.

Other US signs assessed included bile duct dilation, liver morphology, and echogenicity for dysmorphia, portal hypertension, and polysplenia syndrome (anomalies detected in situ, asplenia, polysplenia, interruption of the retro-hepatic inferior vena cava, and preduodenal portal vein). Clinical symptoms and signs were reported and presented in the results alongside US parameters determining the presence or absence of BA based on proposed cutoff points (Table S1).

### BA diagnosis

**Figure 1.**
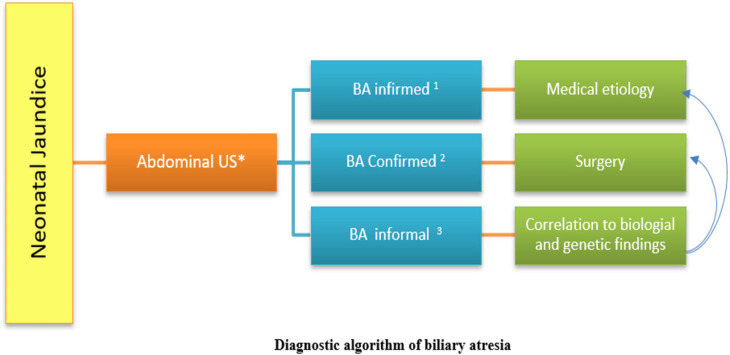
Diagnostic algorithm of biliary atresia (BA). *Abdominal ultrasound done after 10 hours of fasting by the same operator. Signs to identify (small or not detected gallbladder (GB), It would be cord sign, alteration of the evacuation of the GB). ^1^No signs of BA. ^2^All signs of BA are present. ^3^Few signs.

When a neonate presented with jaundice, abdominal ultrasonography was performed to assess the biliary structure, with three possible outcomes: BA ruled out, confirmed, or inconclusive (Figure 1).

1. BA ruled out: If the US suggested an etiology other than BA, further diagnostic evaluation was performed to identify and manage the underlying cause of jaundice.
2. BA confirmed: When US confirmed BA, the patient was prepared for surgery to verify the diagnosis and address the atresia.
3. BA inconclusive: If US findings suggested BA but were inconclusive, the patient underwent further diagnostic evaluations, including biological and genetic assessment. If BA was confirmed, surgery was scheduled; if another anomaly was found, appropriate medical treatment was initiated.

Integrating clinical, laboratory, and US findings ensured a thorough approach to diagnosing and managing jaundiced neonates. This method optimized patient outcomes while preventing unnecessary surgical interventions.

**Figure 2.**
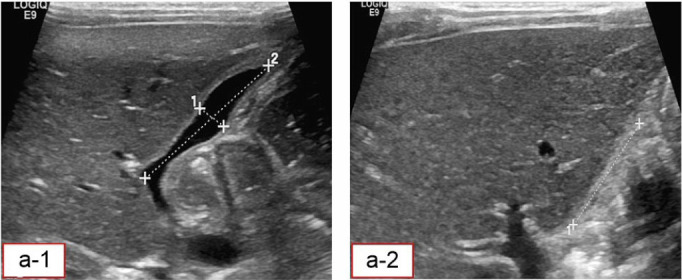
Ultrasound image of a normal GB with normal contraction. a-1, GB length (2) and width (1) after 10 hours fasting. a-2, GB length after drinking a bottle of milk, and depicts the collapsed width.

**Figure 3.**
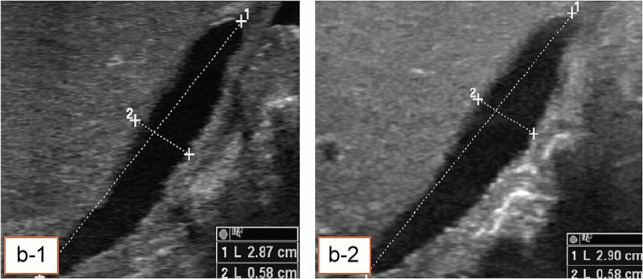
Ultrasound image of an atretic GB. b-1, length (1) and width (2) after 10 hours of fasting. b-2, the same GB after drinking a bottle of milk. No difference in measurements was observed, suggesting GB abnormality—biliary atresia.

**Figure 4.**
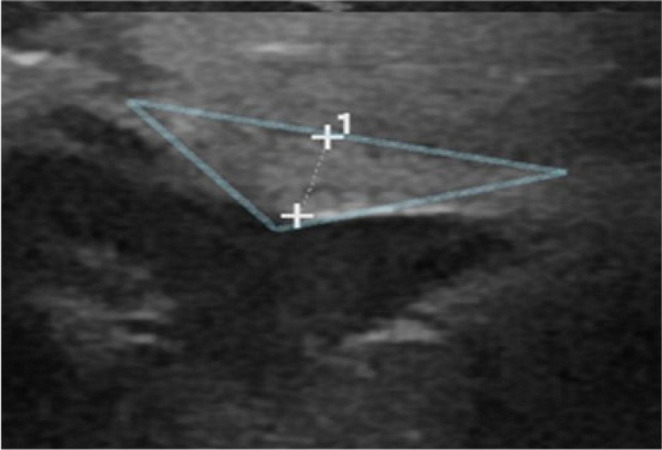
Cord sign: a triangular hyperechoic layer in the hepatic hilus.

**Figure 5.**
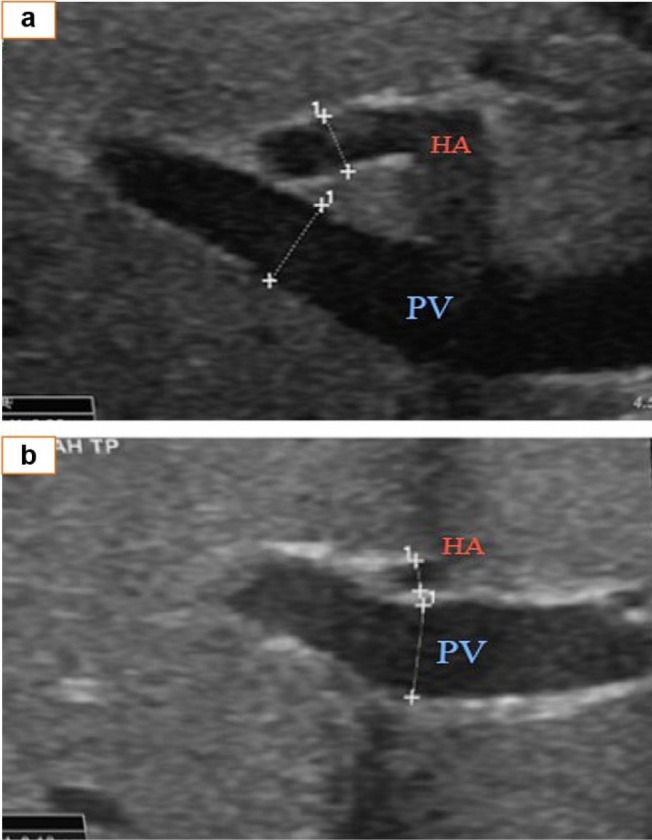
Ultrasound indicating hepatic and portal vein measurements. a, The HA/PV ratio is 0.25/0.38. At approximately 0.66, this ratio is >0.49, suggesting no BA. b, The HA/PV ratio is 0.13/0.39. At approximately 0.33, this ratio is <0.49 and suggests BA. HA: hepatic artery, PV: portal vein, d1: the width of the HA, d2: the width of the PV.

Sixty-one jaundiced newborns and infants were admitted and divided into two groups based on clinical, laboratory, and US findings. Group 1 included 35 patients diagnosed with BA (either by US or other methods), and Group 2 comprised 24 patients diagnosed with other biliary conditions. Two cases were inconclusive. Surgical intervention confirmed BA in 23 patients. Patients with a negative BA diagnosis based on US who did not undergo surgery were closely followed up. Those who underwent surgery were diagnosed with BA, while those who did not were confirmed as non-BA. All patients were carefully evaluated. Figures 2 to 5 present the samples of ultrasound images taken from the patients.

### Data analysis

SPSS software (SPSS, version 23.0; SPSS, Chicago, IL) was used for statistical analysis. Frequencies and percentages were calculated for categorical variables, while means and standard deviations were calculated for continuous variables. The *χ*^2^ statistical test of choice was employed for categorical variables, and the independent ttest for continuous variables. The diagnostic characteristics of US performance were expressed as sensitivity and specificity. Cutoff values for quantitative variables were determined from the receiver operating function curve (data not shown). The significance was set at a *p* value of 0.05.

### Ethics

The Medical Research Centre and institutional review board (IRB) of the Faculty of Medicine at Manastir approved this study (approval no. IORG 00097838 N184 OMB 0990-0279). The study was conducted following the standards outlined in the Helsinki Declaration of 1975. This essential phase guarantees that the study conforms to ethical standards, safeguarding participant rights and wellbeing. A favorable review was anticipated, and any adjustments suggested by the IRB were diligently implemented to meet the specified guidelines and criteria.

## RESULTS

### Descriptive statistics

US examination was conducted on 61 newborns and infants aged 6 to 160 postnatal days (58.28 ± 34.24); 60.7% were males (n = 37). Definitive BA was diagnosed in 32 patients, most of whom (42.6%; n = 26) had Ohi type 1 BA. The GB was not detected by the US in 20 patients. Surgery was carried out for 67.2% (n = 41) of the patients. The clinical and US characteristics of the patients are presented in Tables S2 and S3.

The US diagnosis of BA revealed that 39.3% (*n* = 24) of cases showed no signs in favor of BA, 57.4% (*n* = 35) had an appearance suggestive of BA, and 3.3% (*n* = 2) were nonconclusive. Overall, 52.5% (*n* = 32) of the total cases were diagnosed with BA, while 47.5% (*n* = 29) were not. Regarding surgical intervention, 67.2% (*n* = 41) of the patients underwent surgery, whereas 32.8% (*n* = 20) did not. The data indicates that out of the total cases reviewed, 52.5% were diagnosed with BA either through US or surgical exploration. The US results were categorized into three groups: 39.3% showed no signs of BA, 57.4% had an appearance suggestive of BA, and 3.3% were nonconclusive.

### Inferential statistics

The association between clinical and individual US parameters was explored. The presence of a cord sign [X^2^ (1, *n* = 61) = 53.52; *p*< 0.001], GB evacuation alteration [X^2^ (1, *n* = 61) = 18.41; *p* < 0.001], and HA/PV ratio were significantly associated with BA diagnosis [X^2^ (1, *n* = 61) = 30.25; *p* < 0.001].

### Sensitivity and specificity results

Sensitivity and specificity were reported for different US findings to determine their suitability for BA diagnosis. The positive cord sign had a sensitivity of 93.8% (95% CI, 79.2%–99.2%) and a specificity of 96.6% (95% CI, 82.2%–99.9%). Evacuation alterations and inability to assess the GB sign had a specificity of 86.2% (95% CI, 68.3%–96.1%) with the same sensitivity of 93.8% (95% CI, 79.2%–99.2%). The HA/PV vein ratio showed a sensitivity of 87.5% (95% CI, 71.0%–96.5%) and a specificity of 86.2% (95% CI, 68.2%–96.5%). Each method alone carried a risk of false positives and negatives. Some patients were misdiagnosed (6% for cord sign and evacuation alteration), and a few patients underwent unnecessary surgery: 3% and 14% for cord sign alone and evacuation alteration alone, respectively (Table 1).

Therefore, different US findings can be combined to achieve sensitivity rates of approximately 100%, with no false negative cases. Having a positive cord sign or the presence of alteration of evacuation indicates the possibility of BA with 100% sensitivity (95% CI, 89.1%–100%) and 86.2% specificity (95% CI, 68.3%–96.1%). However, a combination of evacuation alteration or HA/PV ratio maintains 100% sensitivity but reduces specificity to 75.9% (95% CI, 56.5%–89.7%). The same rate of 100% sensitivity is obtained with the presence of evacuation alteration or porta hepatic thickness (≥2.4; sensitivity = 100%; 95% CI, 89.1%–100%); however, a specificity of 86.2% (95% CI, 68.3%–96.1%) is observed.

Therefore, using a positive cord sign or the presence of evacuation alteration as a diagnostic tool, or the presence of evacuation alteration or porta hepatic thickness (≥2.4) indicates the possibility of diagnosing BA with 100% sensitivity. However, despite this observation, 13.8% of the results were false positives (Table 2).

Therefore, based on the available data, the US parameter can reasonably be considered to show promising results as a reliable diagnostic tool for suspected BA.

## DISCUSSION

Notably, numerous radiological diagnostic modalities are used to evaluate the anatomical abnormalities of the biliary system, including magnetic resonance cholangiopancreatography, percutaneous cholecysto-cholangiography, endoscopic retrograde cholangiopancreatography, and hepatobiliary scintigraphy.^7^ However, the US is the preferred method for BA diagnosis because it is a real-time, non-invasive, and radiation-free imaging technique.^11^ Moreover, the US is readily available in most hospitals and is affordable. In addition, cutting-edge US developments allow for the combination of high-frequency US (>10 MHz) for greyscale and color Doppler for optimum spatial resolution.^7,11^ Taken together, neonatal US is a valuable tool for the early detection and diagnosis of BA in newborns with jaundice.

This study provides evidence-based data on the most reliable US parameters for BA diagnosis to reduce surgical intervention risks. The most prevalent US signs significantly correlating with BA are triangular cord signs, anatomical abnormalities of the GB, GB contraction and evacuation alteration, common bile duct visibility, enlarged hepatic artery, cysts, and polysplenia syndrome.^11,12^ In this study, we found a significant correlation between certain US parameters and the presence of BA, confirming the overall accuracy of a US examination in BA detection. We also found that combining the triangular cord and evacuation alteration sign methods yields the highest rates of sensitivity (100%) and specificity (86.2%), compared with other parameters. Furthermore, the same result was obtained by combining evacuation alteration and porta hepatic thickness (≥2.4 mm) methods. Therefore, either of these two combined methods is a reliable predictor of BA and surgical intervention. However, additional diagnostic modalities are required in the absence of such signs.

The triangular cord sign is a US feature that identifies the fibrosed obliterated biliary ducts close to the wall of the anterior right portal vein. In the transverse plane, the echogenic thickening of 3 to 4 mm close to the branching point of the main portal vein can be triangular or tubular. The cord sign is a very crucial BA diagnostic parameter; however, the reported accuracy rates regarding sensitivity and specificity are inconsistent. The triangular cord sign has the highest odds ratio for BA diagnosis compared with the other parameters, exhibiting low sensitivity (68%) and high specificity (95%) rates.^12^ Studies report a specificity of 97% in infants aged <90 days,^14^ and 100% in infants with cholestatic BA aged 2 to 12 weeks.^15^ Therefore, we hypothesized that the accuracy of the triangular cord sign as the only diagnostic parameter may be influenced by neonatal age.^16^ Given the difficulty of identifying atretic biliary ducts in patients with early-stage BA due to the continuous evolution of the condition over time,^17^ we researched a combination of US parameters.

GB abnormalities include its invisibility, length <15 to 19 mm, abnormal shape, and lack of evacuation alteration.^4^ The combination of a triangular cord sign and GB abnormality was 84% sensitive and 100% specific for BA diagnosis.^12^ However, this low sensitivity allows for false positives in 16% of diagnostic cases. To increase the diagnostic power of the triangular cord sign, our study examined the combination of the triangular cord sign with the evacuation alteration sign. The results returned 100% sensitivity, indicating the ability to identify all patients. The specificity was 86%, thus reducing the false negative cases. This synergistic approach improves early diagnosis, allowing for timely intervention and better outcomes for affected infants. Similarly, the combination of GB evacuation alteration and porta hepatis thickness (≥2.4 mm) yields comparable results. Contrary to the literature, the sensitivity of the combinatory diagnostics in this study was higher (100%) with lowered specificity (86.2%), suggesting a more robust diagnosis of all positive cases. This discrepancy with the literature is attributed to the age of the patients in this study, which ranged from 6 to 160 days, with a mean of 58 days, while in the literature, the age gap was smaller. Therefore, the possibility of visualizing a well-developed cord sign may be associated with findings in older patients who do not experience the technical difficulties associated with extremely young patients.^18,19^

According to the literature, the HA/PV ratio was significantly correlated with the diagnosis of BA. In patients with BA, the right hepatic artery was reported to be larger than that in those with hepatitis (median = 1.3 mm,^20^ interquartile range, 1-1.8 mm), and the portal vein diameter was larger (mean, 4.3 ± 0.8 mm) compared with that in those without BA (mean, 3.9 ± 0.8 mm).^21^ Another study reported that the final HA/PV ratio was 0.45 with a 76% sensitivity and 79% specificity.^20^

In our study, an HA/PV ratio of >0.49 was adopted as suggestive of BA. Its sensitivity and specificity are higher than those reported in the literature, 87.5% and 86.2%, respectively. However, despite the 100% sensitivity, the combination of an evacuation alteration sign and HA/PV ratio yields a low specificity of 75.9%. Therefore, although the HA/PV ratio can provide a sensitive diagnosis of BA, it cannot confidently rule out negative cases.

To sum up, while the US is a valuable and accessible initial screening tool for BA, it should be incorporated into a comprehensive diagnostic approach. Combining US findings with additional diagnostic modalities ensures optimal accuracy, facilitating timely and appropriate interventions that are crucial for improving the prognosis of infants with BA.

### Strengths and limitations

The research design is a cohort study that minimizes selection bias and ensures accuracy. However, although statistical analysis identified an optimal combination of sensitivity and specificity, the diagnostic tool did not reach 90% specificity. Therefore, integrating additional diagnostics with the US is recommended to confirm negative cases.

The study's main limitation is the small sample size, which is challenging to address given the rarity of the disease and the need for expertise.

### Recommendations

Recent findings confirm the reliability of the US as a diagnostic tool for BA, making it a cost-effective option for widespread use. However, negative results should be followed by additional tests. Well-structured control studies are crucial to ensure accurate diagnoses and appropriate treatment. Well-structured control studies, standardized US protocols, and enhanced training programs are essential for ensuring diagnostic accuracy, consistency, and appropriate treatment.

## CONCLUSIONS

Timely and accurate diagnosis of BA is essential, as delayed identification significantly impacts patient prognosis and survival outcomes. The US has emerged as a valuable, noninvasive diagnostic modality for cholestatic infants, particularly when specific sonographic markers are present. In this study, the combination of the triangular cord sign and evacuation alteration demonstrated the highest diagnostic accuracy (sensitivity: 100%, specificity: 86.2%), comparable to the combination of evacuation alteration and porta hepatis thickness (≥2.4 mm). Although the US is valuable for BA detection, its 16% false-positive rate highlights its limitation as a standalone diagnostic tool, making it unable to definitively exclude the diagnosis in all cases. Identifying the most sensitive and specific US markers provides evidence-based data to enhance diagnostic strategies, particularly in resource-limited settings. Future research should focus on validating these findings through larger, multicenter studies and exploring the integration of the US with other non-invasive diagnostic modalities.

## STATE OF THE ART

Recent advancements in US technology have greatly enhanced the diagnostic accuracy for congenital BA, a serious neonatal liver condition. While the sensitivity and specificity of individual US parameters in diagnosing BA have remained ambiguous, our study provides evidence-based data highlighting the most reliable US indicators for BA detection, thereby minimizing the need for surgical interventions.

Key US parameters that have shown a strong correlation with BA include the triangular cord sign, anatomic abnormalities of the GB, GB contraction and evacuation alterations, visibility of the common bile duct, enlarged hepatic artery, presence of cysts, and polysplenia syndrome. These findings are substantiated by prior research, reinforcing the significance of these indicators in BA diagnosis.

Our study reveals that combining the triangular cord sign with evacuation alterations yields the highest sensitivity (100%) and specificity (86.2%) compared to other individual parameters. Similarly, the combination of evacuation alterations and porta hepatic thickness (≥2.4 mm) also demonstrates high diagnostic accuracy. These combined methodologies prove to be reliable predictors of BA, suggesting a more efficient, noninvasive diagnostic approach.

However, it is important to note that in the absence of these specific signs, additional diagnostic modalities are required to ensure accurate diagnosis. This highlights the need for ongoing research and development of new diagnostic techniques to further improve the sensitivity and specificity of BA detection, ultimately reducing the risks associated with unnecessary surgical procedures.

By advancing the understanding and application of these US parameters, low-income medical facilities can benefit from cost-effective and accessible diagnostic tools, improving patient outcomes in BA detection and treatment.

### Acknowledgements

The authors sincerely thank the patients and their guardians who participated in this study for their valuable time, cooperation, and trust. Their willingness to contribute to medical research is deeply appreciated.

### Funding

This research did not receive any specific grant from the public, commercial, or not-for-profit funding agencies.

### Declarations

Compliance with ethical standards: The patients provided informed consent for data submission and publication. The Medical Research Centre, Faculty of Medicine at Manastir, confirmed consent, data anonymization, and approved publication.

### Data availability statement

Data will be made available on request.

### List of abbreviations

**Table:** 

BA	biliary atresia
GB	gall bladder
HA/PV ratio	hepatic artery-to-portal vein ratio
IRB	institutional review board
TCT	triangular cord thickness
US	ultrasound

### Conflict of interest

The authors declare no conflicts of interest or financial ties. All co-authors approve the manuscript's content.

### Authors’ contributions

BYS: Conceptualization, Methodology, Investigation, Data Curation, Writing—Original Draft. ANA: Conceptualization, Methodology, Investigation, Data Curation, Writing—Original Draft, Writing—Review and Editing of the final draft. MM, BAAK, MS, BFM, BSR, AM, KA, ZA and SL: Investigation and Data Curation.

## Figures and Tables

**Table 1:** Diagnostic efficacy of different ultrasound findings.

		Biliary atresia							
		No	Yes	Sensitivity (95%CI)			Specificity (95% CI)		
Porta hepatic thickness (mm)	<2.4	28	5	84.4 %	67.2 %	94.7 %	96.6 %	82.2 %	99.9 %
	≥2.4	1	27						
The ratio of the hepatic artery to/portal vein	<0.49	25	4	87.5 %	71.0 %	96.5 %	86.2 %	68.3 %	96.1 %
	≥0.49	4	28						
Hepatic artery (mm)	<2.15	26	9	71.9 %	53.3 %	86.3 %	89.7 %	72.6 %	97.8 %
	≥2.15	3	23						
GB volume (mL)	≥0.23	25	3	90.6 %	75.0 %	98.0 %	86.2 %	68.3 %	96.1 %
	<0.23 or NA	4	29						
GB length (mm)	≥17.5	26	5	84.4 %	67.2 %	94.7 %	89.7 %	72.6 %	97.8 %
	<17.5 or NA	3	27						
GB width (mm)	≥4.5	25	3	90.6 %	75.0 %	98.0 %	86.2 %	68.3 %	96.1 %
	<4.5 or NA	4	29						
Cyst	Absent	28	26	81.3 %	63.6 %	92.8 %	3.4 %	0.1 %	17.8 %
	Present	1	6						
Microcyst	Absent	29	28	87.5 %	71.0 %	96.5 %	0.0 %	0.0 %	11.9 %
	Present	0	4						
Polysplenia	Absent	28	29	90.6 %	75.0 %	98.0 %	3.4 %	0.1 %	17.8 %
	Present	1	3						
Preduodenal PV	Absent	29	31	96.9 %	83.8 %	99.9 %	0.0 %	0.0 %	11.9 %
	Present	0	1						
Cord sign	Negative	28	2	93.8 %	79.2 %	99.2 %	96.6 %	82.2 %	99.9 %
	Positive	1	30						
Alteration of evacuation	No	25	2	93.8 %	79.2 %	99.2 %	86.2 %	68.3 %	96.1 %
	Yes or NA	4	30						

Abbreviations: GB: gall bladder, NA: gallbladder was not present or impossible to assess, PV: portal vein

**Table 2:** Diagnostic accuracy of combined ultrasound findings.

		Biliary atresia							
		No	Yes	Sensitivity (95%CI)			Specificity (95% CI)		
Positive cord sign or presence of alteration of evacuation	No	25	0	100.0%	89.1 %	100.0%	86.2%	68.3%	96.1 %
	Yes	4	32						
Positive cord sign or GB volume (≤0.23 mL or NA)	No	4	2	93.8%	79.2%	99.2%	13.8%	3.9%	31.7%
	Yes	25	30						
Positive cord sign or GB width (≤4.5 mm or NA)	No	4	2	93.8%	79.2%	99.2%	13.8%	3.9%	31.7%
	Yes	25	30						
Presence of alteration of evacuation or porta hepatic thickness (≥2.4 mm)	No	25	0	100.0%	89.1 %	100.0%	86.2%	68.3%	96.1 %
	Yes	4	32						
Presence of alteration of evacuation or ratio hepatic artery/portal vein (≥0.49)	No	22	0	100.0%	89.1 %	100.0%	75.9%	56.5%	89.7%
	Yes	7	32						
Presence of alteration of evacuation or hepatic artery (≥2.15 mm)	No	23	0	100.0%	89.1 %	100.0%	79.3%	60.3%	92.0%
	Yes	6	32						

Abbreviations: GB: gall bladder, NA: gallbladder was not present or impossible to assess, PV: portal vein

## Supplement

Supplementary Tables S1-S3

## References

[1] Liu S, Li T, Yang Q, Ke X, Zhan J. (2024). Biliary atresia: the development, pathological features, and classification of the bile duct..

[2] Lyu H, Ye Y, Wang B. (2023). FIB-4 and APRI scores for progressive liver fibrosis diagnosis in children with biliary atresia..

[3] Dara M, Azarpira N, Motazedian N, Hossein-Aghdaie M, Dehghani SM, Geramizadeh B (2024). Expression of miR-let7b and miR-19b in progressive familial intrahepatic cholestasis (PFIC) children..

[4] Gincu G, Gudumac E, Brani te N, Revenco I, Haidarli D, Samciuc O. (2020). Diagnosis and modern medical-surgical tactics in treatment of biliary atresia in children..

[5] Spivack OKC, Dellenmark-Blom M, Dingemann J, Ten Kate CA, Wallace V, Bramer WM (2024). A narrative review of patient-reported outcome measures and their application in recent pediatric surgical research: advancing knowledge and offering new perspectives to the field..

[6] So K, Shinagawa T, Yoshizato T, Fukahori S, Asagiri K, Maeno Y (2023). Difficulty in the diagnosis of biliary atresia splenic malformation syndrome in utero..

[7] Sandberg JK, Sun Y, Ju Z, Liu S, Jiang J, Koci M (2021). Ultrasound shear wave elastography: does it add value to gray-scale ultrasound imaging in differentiating biliary atresia from other causes of neonatal jaundice?.

[8] Son TN, Mai DV, Tung PT, Liem NT. (2023). Laparoscopic versus open Kasai procedure for biliary atresia: longterm results of a randomized clinical trial..

[9] Zhou W, Chen D, Jiang H, Shan Q, Zhang X, Xie X (2019). Ultrasound evaluation of biliary atresia based on gallbladder classification: is 4 hours of fasting necessary?.

[10] Zhou LY, Wang W, Shan QY, Liu BX, Zheng YL, Xu ZF (2015). Optimizing the US diagnosis of biliary atresia with a modified triangular cord thickness and gallbladder classification..

[11] Zhou W, Zhou L. (2021). Ultrasound for the diagnosis of biliary atresia: from conventional ultrasound to artificial intelligence.. https://doi.org/10.3390/diagnostics12010051.

[12] Napolitano M, Franchi-Abella S, Damasio MB, Augdal TA, Avni FE, Bruno C (2021). Practical approach to imaging diagnosis of biliary atresia, Part 1: prenatal ultrasound and magnetic resonance imaging, and postnatal ultrasound..

[13] Van Heerden Y, Maher H, Etheredge H, Fabian J, Grieve A, Loveland J (2019). Outcomes of paediatric liver transplant for biliary atresia..

[14] Mittal V, Saxena AK, Sodhi KS, Thapa BR, Rao KL, Das A (2011). Role of abdominal sonography in the preoperative diagnosis of extrahepatic biliary atresia in infants younger than 90 days..

[15] Tan Kendrick AP, Phua KB, Ooi BC, Tan CE. (2003). Biliary atresia: making the diagnosis by the gallbladder ghost triad..

[16] Saxena AK, Mittal V, Sodhi KS. (2012). Triangular cord sign in biliary atresia: does it have prognostic and medicolegal significance?.

[17] Brahee DD, Lampl BS. (2022). Neonatal diagnosis of biliary atresia: a practical review and update..

[18] He M, Xie H, Du L, Lei T, Zhang L. (2022). Postnatal outcomes of fetuses with isolated gallbladder anomalies: be aware of biliary atresia..

[19] Ho A, Sacks MA, Sapra A, Khan FA. (2021). The utility of gallbladder absence on ultrasound for children with biliary atresia..

[20] Kim WS, Cheon JE, Youn BJ, Yoo SY, Kim WY, Kim IO (2007). Hepatic arterial diameter measured with US: adjunct for US diagnosis of biliary atresia..

[21] Choochuen P, Kritsaneepaiboon S, Charoonratana V, Sangkhathat S. (2019). Is “gallbladder length-to-width ratio” useful in diagnosing biliary atresia?. https://doi.org/10.1016/j.jpedsurg.2019.01.008.

