# Supplementary material for: Ultrasound reliability for biliary atresia diagnoses in children: a single-center study
Source: Qatar Med J. 2025 Aug 17;2025(3):68. doi: 10.5339/qmj.2025.68 (PMC12894349; doi:10.5339/qmj.2025.68)
Supplement: Supplementary Tables S1-S3 [file qmj-2025-03-68-s001.pdf]

Supplement

Table S1: Data collected either by ultrasound or clinically.

Data collected	Values with biliary atresia	Comment
**Patient age**	in days	
**Sex**	boy and girls	
**Cut off - porta hepatic thickness**	≥2.4mm	
**Cut off - ratio hepatic artery/portal vein**	> 0.49	
**Cut off hepatic artery**	> 2.15	
**Cut off GB volume**	≤ 0.23	when the gallbladder was absent, the value was recorded as zero
**Cut off GB length**	≥ 17.5	when the gallbladder was absent, the value was recorded as zero
**Cut off GB width**	≤ 4.5	when the gallbladder was absent, the value was recorded as zero
**Cyst**	absence	
**Microcyst**	absence	
**Polysplenia**	absence	
**Predudenal portal vein**	absence	
**Cord sign**	detected	
**Evacuation of gb after meal**	poor evacuation	
**Main biliary duct**	not dilated	
**Intrahepatic bile ducts**	not dilated	
**Jaundice**	present	
**Clay stool**	present	
**Hepatomegaly**	present	
**Portal hypertension**	present	
**Splenomegaly**	present	
**Ascites**	present	
**Collateral veinous circulation**	present	
**Classification**	OHI classification of BA	

Table S2: Correlation between clinical and individual US parameters and final diagnosis of biliary atresia.

Parameter	N	Statistic*	P	Significance
Cut off PH	61	36.93	<0.001	****
Cut off Ratio HAPV	61	30.25	<0.001	****
Cut off HA	61	21.102	<0.001	****
Cut off V	40	13.611	<0.001	***
Cut off Length	40	9.858	<0.01	**
Cut off width	40	13.611	<0.001	***
Cyst	61	2.162	0.141	ns
Microcyst	61	2.108	0.147	ns
polysplenia	61	0.173	0.677	ns
Préduodenal PV	61	0	1	ns
Cord sign	61	53.515	<0.001	****
Alteration of evacuation	41	18.401	<0.001	****
Liver	61	8.679	<0.01	**
Jaundice	61	0.148	0.701	ns
Stool	61	17.091	<0.001	****
Hepatomegaly	61	5.854	<0.05	*

Portal hypertension	61	4.195	<0.05	*
Splenomegaly	17	0	1	ns
Ascites	17	0.501	0.479	ns
Collateral Veinous circulation	17	0.354	0.552	ns
Main biliary duct	61	2.741	0.0978	ns
Intrahepatic bile ducts	61	0.625	0.429	ns

Note 1:*Chi square test. N: number of patients

Table S3: Correlation of US parameters and the final diagnosis of biliary atresia.

Parameter	P-value	Lower CI	Upper CI	Significance
Width-gallbladder	<0.001	2.2094	5.1954	***
Length-gallbladder	<0.05	1.9106	13.0894	***
Volume-gallbladder	<0.001	0.5779	1.3853	***
RatioL_W	<0.01	0.6552	2.725	***
Hepatic_artery	<0.001	-0.8993	-0.4386	***
Portal_vein	0.534	-0.3318	0.6331	NS
Ratio_HA_PV	<0.001	-0.2939	-0.1517	***
Plaque	<0.001	-2.8153	-1.5509	***

Note 2:*t test. CI: confidence interval.
